# Lifetime prevalence and treatment of mental disorders in Saudi youth and adolescents

**DOI:** 10.1038/s41598-023-33005-5

**Published:** 2023-04-15

**Authors:** Yasmin Altwaijri, Alan E. Kazdin, Abdullah Al-Subaie, Abdulhameed Al-Habeeb, Sanaa Hyder, Lisa Bilal, Mohammad Talal Naseem, Edward De Vol

**Affiliations:** 1 King Salman Center for Disability Research, Riyadh, Saudi Arabia; 2 Biostatistics, Epidemiology and Scientific Computing Department, King Faisal Specialist Hospital and Research Centre, MBC 03, PO Box 3354, Riyadh, 11211 Saudi Arabia; 3 SABIC Psychological Health Research & Applications Chair (SPHRAC), College of Medicine, King Saud University, Riyadh, Saudi Arabia; 4 Department of Psychology, Yale University, New Haven, CT USA; 5 Edrak Medical Center, Riyadh, Saudi Arabia; 6 National Center for Mental Health Promotion, Ministry of Health, Riyadh, Saudi Arabia; 7 Health and Meaningful Dialogue (HAMD) Centre, Manchester, UK

**Keywords:** Psychology, Diseases, Risk factors

## Abstract

Previous global and regional studies indicate that adolescents and young adults (i.e., youth) are affected by various mental disorders with lifelong consequences. However, there are no national estimates of mental disorders prevalent among Saudi youth. Using data from the Saudi National Mental Health Survey (SNMHS), we examined the lifetime prevalence, treatment, and socio-demographic correlates of mental disorders among Saudi youth (aged 15–30). A total of 4004 interviews were conducted using the adapted Composite International Diagnostic Interview (CIDI 3.0). Cross tabulations and logistic regression were used to generate estimates for the SNMHS youth sample (n = 1881). The prevalence of a mental disorder among Saudi youth was 40.10%, where anxiety disorders affected 26.84% of the sample, followed by disruptive behavior disorders (15.44%), mood disorders (9.67%), substance use disorders (4%) and eating disorders (7.06%). Sex, education, parental education, income, marital status, region, and family history of disorders were significant correlates of various classes of mental disorders. Only 14.47% of Saudi youth with any mental disorder received treatment for a lifetime disorder. Age, parental education, and family history of disorders emerged as significant correlates of mental health treatment. Lifetime mental disorders are highly prevalent among Saudi youth. There is an unmet need for culturally sensitive and age-appropriate treatment of lifetime mental disorders among youth in Saudi Arabia.

## Introduction

A significant percentage of adolescents worldwide are affected by mental disorders^1,2^. The World Health Organization (WHO) defines adolescence as the age between 10 and 19 years, while youth refers to those between 15 and 24 years^3^. In this paper, we refer to adolescents and young adults in the age range between 15–30 years as youth, bearing in mind that this age category has been previously defined in different ways, such as 18–34^4^, 15–29^5^, and 14–35^6^. This time period is characterized by dynamic brain development as an outcome of individuals' interaction with their social environment^7^. When youth do not acquire optimal physical, cognitive, emotional, and economic resources during their early years, problems related to emotional control and behavior typically emerge^3^. This is corroborated by global epidemiological studies^8,9^, which suggest that most mental disorders first onset early in life, i.e., during childhood and adolescence. Additionally, young people in this age group have the worst access to and engagement with mental health services compared to other age groups^8,10^.

During adolescence, individuals have low resistance to peer influences, low levels of future orientation, and low risk perception, which is linked to risk-taking behavior, and poor self-regulation^3,11^. Psychologically, it is a time of identity formation, where school and family environments become crucial contexts; parental characteristics like education level and marital status (e.g. divorce) become pertinent, potentially impacting the mental health status of children in their mid-adolescence and early adulthood^12^; later, critical life changes are related to the individuals’ career and parenthood^13–15^. Indeed, when psychological distress during the early years is not mitigated, resulting mental disorders affect the individuals' motor, cognitive, learning and behavioral development with lifelong consequences; these outcomes include functional impairment, poor quality of life, and increased risk of later medical and comorbid psychiatric disorders^10,16,17^. For example, the risk of having at least 1 mental disorder by the age of 16 years is significant, especially for girls whose chances of continuing to have a mental disorder, or of developing another episode after remission are much higher than those of their unaffected peers^18^.

Similar prevalence and treatment estimates for mental disorders in youth are reported in the Arab region^19–22^, in countries like Oman^23,24^, Jordan^25,26^, United Arab Emirates^27^, and Lebanon^28^. In fact, mental health and substance use problems account for more than half of the high ranking disability-adjusted life years (DALYs) among adolescents in high income countries including the Gulf Cooperation Council (GCC)^29,30^. Although there are distinct socioeconomic diversities between the countries in the Arab region, they share important similarities with respect to language, religion, culture, and demographics, which can be instrumental in shaping mental health related perceptions and management of psychiatric disorders^31,32^. Still, individual characteristics and contextual differences play an important role^20^; structural determinants such as unemployment, military conflicts, changing family environments, changing gender roles, increased education and age at marriage, rise of social media, tension between tradition, attachment to family, community and country are some of the strong determinants of mental health among Arab youth^25,32,33^.

The Kingdom of Saudi Arabia (KSA) has a population of over 34 million inhabitants^34^. According to the 2020 Saudi population survey, more than a quarter (> 24%) of the population was between 15 and 30 years of age^35^; and the estimated median age was 25 years old^36^. Among school-going youth, regional Saudi studies indicate that some specific mental disorders are highly prevalent^37–39^, and among girls^40–43^, and boys^44,45^ individually. Regarding national estimates, studies have found symptoms of depression and anxiety prevalent among adolescents aged 10–19^46,47^ with feelings of sadness or hopeless, and worry more prevalent among females and older adolescents^48^. Moreover, according to the Saudi National Mental Health Survey (SNMHS)—a nation-wide general population psychiatric epidemiological study with a representative household sample of Saudis aged 15–65—various mental disorders have their onset during adolescence as well as in early adulthood^49^. Despite the wide-ranging literature on this topic, national estimates of various mental disorders prevalent among the Saudi youth—part of the general population as opposed to samples from schools in previously mentioned studies—are unknown.

While young Saudis are reported to have a favorable attitude towards seeking help for their mental health^50^, there are also no national estimates pertaining to treatment service use for mental disorders among Saudi youth. Largely, research indicates that despite worrying prevalence patterns, mental health needs of youth in developed countries^51^, and Arab countries^20,24,52^ including the KSA^29^ are poorly met. This has immediate effects and long-term negative implications on the wellbeing of the affected youth as well as their families, further impacting future generations and society as a whole^25^. Yet research in this field continues to be underfunded^53^, and inadequate^29^. Data-driven estimates are needed to inform, develop, and refine the existing mental health services and policies for youth in most countries from the Eastern Mediterranean region^54^, including the KSA. This information is necessary to establish resource allocation priorities for planning, designing and implementing intervention programs for Saudi youth, which will improve the prevention, diagnosis and treatment of mental disorders. Using the high quality comprehensive dataset from the SNMHS, the goal of this article is to present national estimates related to lifetime prevalence, treatment and socio-demographic correlates for *Diagnostic and Statistical Manual* (DSM)-IV mental disorders among Saudi youth in the KSA.

## Methods

### Sample

In line with the methods of the World Mental Health Survey Initiative^55^, the SNMHS used a multistage clustered area probability sampling design in which Primary Sampling Units were established separately within each of the country’s administrative (barring Jazan & Najran due to political conflict) based on census counts from maps provided by the Ministry of Economy and Planning in order to nationally represent Saudi citizens between the age 15–65. After being selected from a household listing interview in which interviewers made am in-person visit to each household, introduced the study to a household member, and collected a listing of all non-institutionalized, ambulatory Arabic-speaking Saudi nationals aged 15–65 as potential respondents, the chosen respondent(s) completed Part I of the interview, a component which assessed core disorders. A sub-sample of respondents at this stage—who met lifetime criteria for any of these disorders—were then administered Part II of the interview; this component assessed other selected disorders and correlates. From the final sample of 4,004 interviews, 1,811 youths completed the Part I interview, and 971 youths were administered the Part II interview, with the response rate from youth in the SNMHS being 74.53% (Response rate including no attempt cases: (Number of completed interviews by youth)/(Total sample including completed interviews, no contact or refusals from youth − non-sample cases) = 1811/(2430 − 0) = 0.7453 = 74.53%). We used weights for Part II respondents who did not meet criteria for a core Part I disorder to the inverse of their probability of selection into Part II in order to adjust under-sampling of non-cases and balance the prevalence estimates between the Parts I and II samples. Further details of sampling and weighting procedures can be found elsewhere^56^.

Table 1 presents the weighted characteristics of the study sample. About half of the youth (50.05%) were male. The mean age was 22.41 years (SD = 4.75), with a greater proportion of youth aged 15–22 years (55.17%). The majority of the youth in the sample were students. In terms of parent’s education, 39.79% had low levels of education; and most of the youth came from households with low income (41.83%; the levels of education and income are described later). The sample comprised predominantly of those who were never married (76.87%), lived with their parents (95.61%), in urban areas (82.32%), in the Central region of the Kingdom (33.46%), and did not have a family history of disorders (60.04%).

**Table 1:** Socio-demographic distribution of the youth sample, un-weighted and weighted.

	Un-weighted			Weighted		
	N	%	SE	N	%	SE
Sex						
Female	973	53.73	1.17	973	49.95	1.82
Male	838	46.27	1.17	838	50.05	1.82
Age						
15–22	926	51.13	1.18	926	55.17	1.79
23–30	885	48.87	1.18	885	44.83	1.79
Education						
Low	81	4.47	0.49	81	4.30	0.63
Low average	88	4.86	0.51	88	4.09	0.65
High average	564	31.14	1.09	564	29.80	1.64
High	224	12.37	0.77	224	12.20	1.27
Student	854	47.16	1.17	854	49.60	1.82
Parents' education						
Low	750	41.41	1.16	750	39.79	1.76
Low average	323	17.84	0.90	323	16.88	1.30
High average	439	24.24	1.01	439	24.83	1.56
High	299	16.51	0.87	299	18.50	1.54
Household income						
Low	762	42.08	1.16	762	41.83	1.76
Low average	209	11.54	0.75	209	11.58	1.21
High average	283	15.63	0.85	283	18.39	1.51
High	557	30.76	1.08	557	28.21	1.63
Marital status						
Married	560	30.92	1.09	560	20.22	1.30
Separated/divorced/widowed	45	2.48	0.37	45	2.91	0.66
Never married	1206	66.59	1.11	1206	76.87	1.41
Living with parents*						
Lived with parents	894	93.42	0.80	894	95.61	0.88
One or both parent died	21	2.19	0.47	21	1.73	0.57
Parents divorced	35	3.66	0.61	35	2.31	0.65
Parents never lived together/foster care/others	7	0.73	0.28	7	0.36	0.19
Urbanicity						
Rural	286	15.79	0.86	286	17.68	1.49
Urban	1525	84.21	0.86	1525	82.32	1.49
Region						
Central	587	32.41	1.10	587	33.46	1.69
Eastern	234	12.92	0.79	234	18.58	1.57
Northern	288	15.90	0.86	288	7.57	0.78
Southern	194	10.71	0.73	194	10.39	0.91
Western	508	28.05	1.06	508	30.00	1.71
Family history of disorders*						
Yes	378	45.32	1.72	378	39.96	2.88
No	456	54.68	1.72	456	60.04	2.88

Part I sample was used (n = 1811); *This was asked to only part II sample (n = 971).

Missing data: living with parents = 14; family history of disorders = 137.

### Procedures

Face-to-face interviews were carried out with selected respondents by trained and certified lay interviewers between 2014 and 2016, with pauses for Ramadan and the summer (due to very high temperatures). All interview and training materials were adapted and translated for the Saudi population using evidence-based protocols^57,58^. Details of training, field and quality control procedures have been recorded previously^55,59–61^.

Prior to each interview, all respondents provided signed informed consent, except those below 18 years of age in which case, the consent was given by a parent and/or legal guardian, and the youth gave their assent to be interviewed. Interviews with SNMHS youth lasted approximately two hours and twenty minutes on average. Furthermore, given that sometimes family members requested to stay throughout the course of the interview, in order to minimize the risk of underreporting bias, certain sensitive sections of the survey (incl. substance use disorders) were administered via audio computer-assisted self-interviewing (ACASI) mode which allows respondents to self-report answers directly into a computer without other people present knowing the nature of the questions^59^. Respondents received monetary incentives (worth 100 Saudi Riyals/27 US dollars) for their participation. The study was performed in line with the principles of the Declaration of Helsinki. All survey procedures were approved by the Institutional Review Board at the King Faisal Hospital and Research Center, Riyadh.

### Diagnostic assessment

The instrument used for the SNMHS was the adapted WHO Composite International Diagnostic Interview (CIDI) 3.0^58,59^, a fully structured interview^62^ that generated diagnoses based upon the DSM-IV^63^ in the SNMHS. These CIDI-related diagnoses showed good concordance with diagnoses based on blinded clinician reappraisal interviews published earlier^64,65^. The assessed disorders were grouped into classes such as anxiety disorders (i.e., panic disorder, agoraphobia without panic disorder, social phobia, generalized anxiety disorder, post-traumatic stress disorder, obsessive–compulsive disorder, and separation anxiety disorder), mood disorders (i.e., major depressive disorder, bipolar I and II disorder), eating disorders (i.e., anorexia nervosa, bulimia nervosa, binge-eating disorder), disruptive behavior disorders (i.e., attention deficit hyperactivity disorder (ADHD), conduct disorder, oppositional-defiant disorder, intermittent explosive disorder), and substance use disorders (i.e., alcohol and drug abuse and dependence). Organic exclusion rules and hierarchy rules for all diagnoses have been documented elsewhere^49^.

### Assessment of treatment service use

Part II respondents were asked questions related to treatment obtained in the past 12 months from various professionals. These measures assessed treatment across the healthcare sector and the non-healthcare sector. The healthcare sector comprised of treatment from two main sectors—the general medical sector (family physicians, general practitioners, and other medical doctors, such as cardiologists or gynecologists-urologists, nurses, occupational therapists, and other general healthcare professionals) and the mental health specialty sector (psychiatrists, and other mental health professionals such as psychologists, counselors, psychotherapists, mental health nurses, and social workers). Non-healthcare sector comprised of human services (including social workers or counselors in any setting other than a specialty mental health setting, and religious advisors), and complementary-alternative medicine (including internet use, self-help groups, other healer, such as an herbalist, a chiropractor, or a spiritualist, and other alternative therapy).

Lifetime treatment was measured by administering a smaller number of treatment questions at the end of each diagnostic section when respondents are asked whether they ever in their life saw a medical doctor or any other professional about the disorder assessed in that section of the interview and, if so, their age when they first sought treatment for the disorder. Responses to these disorder-specific questions and the more general treatment questions were combined in our descriptive analyses of treatment prevalence but only disorder-specific responses were used to make projections of eventual lifetime treatment^66^.

### Assessment of sociodemographic correlates

The correlates included age-at-interview, sex, education, marital status, and household characteristics (region, urbanicity, and income). Age-at-interview was divided into two cohorts (15–22, and 23–30). Education was divided into 4 categories of low (0–6 years of education, i.e., completion of primary school), low-average (7–9 years, i.e., completion of secondary school), high-average (10–15 years, i.e., completion of high school, and first 3 years of college), and high (16 + years, i.e., completion of college, and further higher education). Marital status was categorized as never married, married, or previously married (i.e., separated, divorced, or widowed).

Household regions were categorized as Central (Riyadh, Al Qaseem, Hail), Western (Makkah, Al-Madinah), Eastern (Eastern Province), Northern (Northern Frontiers, Al-Jouf, Tabouk), and Southern (Aseer, Al-Baha) regions based on the distribution of administrative areas in the KSA^56^. Urbanicity of a household was coded as either urban or rural. These variables were classified as per the 2010 Saudi Census, General Authority for Statistics. The household income was calculated using the total family household income, respondent’s income and spouse’s income to generate the ‘income per capita’ for each household; this was further divided by the median of income per capita of the total sample (N = 4004) to create the income variable that was categorized as low, low-average, high-average, and high.

Parental characteristics such as parents' education, living with parents, and family history of mental disorders were also assessed. The youth were asked about their parents’ education level, which was grouped like the education categories of the youth; the educational attainment of the parent with the highest level of education was used. The influence of the family constellation was examined by considering whether or not the youth lived with both their parents, lived with parents who were divorced, had one or both parents who had died, never lived with parents, lived in a foster home or any other situation. The presence or absence of family history of any mental disorders was endorsed by the respondent during various sections of the interview.

### Analyses

The data were weighted to adjust for within-household and between-household probabilities of selection differences, discrepancies between sample and population distribution due to random error, and differential response across segments of the population defined by census population variables^59^. All statistical analyses were carried out using SAS Enterprise Guide 9.2 (SAS Institute, Cary, NC). Cross tabulations were used to generate sample characteristics, lifetime prevalence of mental disorders and treatment service use estimates using the PROC SURVEYFREQ procedures. Standard errors of prevalence estimates were reported across sexes, and the two age cohorts. Correlates of prevalence and treatment were examined using logistic regression analysis, following the PROC LOGISTIC procedures. Correlates included age-at-interview, sex, education, marital status, region, urbanicity, household income, parents' education, living with parents, and family history of mental disorders. All socio-demographic variables were included in the regression models simultaneously, and the odd ratios with 95% confidence intervals were reported. The calculation of multivariate significance tests was done with Wald Chi-square tests based on coefficient variance–covariance matrices adjusted for design. Statistical significance was considered at a significance level of 0.05 with two-sided tests.

## Results

### Lifetime prevalence

The prevalence of a DSM-IV mental disorder among Saudi youth was 40.10% (n = 1811) (Table 2). Three significant sex differences were found in disorder-specific prevalence, with higher prevalence among females than males. These included two anxiety disorders—social phobia (8.87% vs. 5.08%, χ^2^ = 4.69, df = 1, p = 0.0320), and obsessive–compulsive disorder (5.59% vs. 2.59%, χ^2^ = 8.29, df = 1, p = 0.0046),—and major depressive disorder (8.78% vs. 3.24%, χ^2^ = 12.45, df = 1, p = 0.0006). However, there was no significant sex difference in overall prevalence of having any lifetime disorder (40.22% vs. 39.97%, χ^2^ = 0.00, df = 1, p = 0.9634). Lifetime prevalence varied significantly with respondent age-at-interview for only one disorder, post-traumatic stress disorder (χ^2^ = 8.99, df = 1, p = 0.0032), with lower rates among the younger age group (aged 15–22, 1.39%) than the older respondents (4.62%).

**Table 2:** Lifetime prevalence of DSM-IV/CIDI disorders, overall and stratified by sex and age in SNMHS youth.

Disorder	Lifetime prevalence			Sex							Age						
				Female			Male			Chi-square	15–22			23–30			Chi-square
	N	%	SE	N	%	SE	N	%	SE		N	%	SE	N	%	SE	
Anxiety disorder																	
Panic disorder^1^	26	1.33	0.31	21	1.90	0.49	5	0.76	0.37	3.35	10	1.12	0.42	16	1.60	0.47	0.56
Generalized anxiety disorder^1^	23	1.73	0.55	17	2.77	1.05	6	0.68	0.30	3.67	7	0.97	0.43	16	2.66	1.12	1.94
Social phobia^1^	107	6.97	0.89	71	8.87	1.29	36	5.08	1.22	4.69*	61	7.63	1.27	46	6.16	1.07	0.89
Agoraphobia^1^	39	2.38	0.57	32	3.47	0.91	7	1.30	0.79	3.19	20	2.42	0.82	19	2.34	0.72	0.01
Post-traumatic stress disorder^2^	54	2.83	0.55	39	3.18	0.73	15	2.48	0.86	0.39	17	1.39	0.46	37	4.62	1.00	8.99*
Separation anxiety disorder^2^	175	14.69	2.40	108	13.94	2.04	67	15.44	3.60	0.20	84	12.49	2.58	91	17.42	3.69	1.21
Obsessive–compulsive disorder^2^	74	4.09	0.67	47	5.59	0.98	27	2.59	0.67	8.29*	40	4.68	1.26	34	3.36	1.15	0.40
Any anxiety disorder^2^	340	26.84	2.80	220	29.43	2.83	120	24.25	4.36	1.24	166	24.28	3.28	174	30.02	3.39	2.00
Mood disorder																	
Major depressive disorder^1^	115	6.01	0.73	85	8.78	1.41	30	3.24	0.70	12.45**	53	4.44	0.76	62	7.94	1.35	4.56*
Bipolar I and/or II^1^	58	3.66	0.72	31	2.82	0.67	27	4.50	1.18	1.66	35	5.15	1.18	23	1.83	0.48	6.98*
Any mood disorder^1^	173	9.67	1.00	116	11.61	1.62	57	7.74	1.23	3.78	88	9.59	1.33	85	9.78	1.38	0.01
Disruptive behavior disorder																	
Conduct disorder^2^	35	2.19	0.48	14	2.28	0.70	21	2.09	0.53	0.05	18	2.30	0.91	17	2.05	0.62	0.04
ADHD^2^	120	11.29	1.76	63	7.73	1.51	57	14.85	3.20	3.69	69	12.28	2.50	51	10.06	1.90	0.63
Intermittent explosive disorder^2^	51	4.73	0.94	24	3.73	1.25	27	5.73	1.34	1.23	29	4.87	1.17	22	4.57	1.42	0.03
Any disruptive behavior disorder^2^	174	15.44	1.81	92	12.90	2.12	82	17.99	3.13	1.65	94	15.86	2.63	80	14.93	1.93	0.10
Substance use disorder																	
Alcohol abuse^2^	1	0.02	0.02	1	0.04	0.04	0	–	–	–	0	–	–	1	0.05	0.05	–
Alcohol dependence^2^	7	0.49	0.23	0	–	–	7	0.97	0.46	–	2	0.24	0.22	5	0.79	0.43	1.36
Drug abuse^2^	39	2.94	0.87	16	1.71	0.48	23	4.16	1.65	2.15	23	3.97	1.50	16	1.65	0.67	1.80
Drug dependence^2^	11	0.60	0.22	6	0.75	0.37	5	0.45	0.23	0.50	4	0.47	0.28	7	0.76	0.35	0.43
Any substance use disorder^2^	55	4.00	0.89	23	2.51	0.59	32	5.49	1.66	3.04	28	4.66	1.54	27	3.17	0.91	0.59
Eating disorder																	
Anorexia^2^	4	1.23	1.07	0	–	–	4	2.45	2.12	–	3	2.04	1.93	1	0.22	0.22	0.88
Binge eating disorder^2^	46	3.17	0.64	30	3.33	0.87	16	3.01	0.92	0.07	21	2.73	0.77	25	3.71	1.04	0.57
Bulimia^2^	43	3.49	0.85	23	3.34	1.00	20	3.64	1.36	0.03	17	3.26	1.20	26	3.77	1.19	0.09
Any eating disorder^2^	85	7.06	1.36	51	6.41	1.28	34	7.72	2.24	0.28	36	7.41	2.13	49	6.64	1.48	0.09
Any^2^	501	40.10	2.94	306	40.22	3.67	195	39.97	4.31	0.00	253	40.28	3.58	248	39.88	3.72	0.01

Part I sample, those who completed the survey (age 15–30) = 1811; Part II sample, those who completed the survey (age 15–30) = 971.

^1^Part I sample, prevalence calculated using part I weights.

^2^Part II sample, prevalence calculated using part II weights.

*p < 0.05; **p < 0.001.

### Sociodemographic correlates

Females were significantly less likely than males to have disruptive behavior disorders and substance use disorders (Table 3). Those who reported low education had significantly high risk of disruptive behavior, and substance use disorders compared to those who were students at the time. Low average education was also associated with significantly higher rate of mood disorders, and substance use disorders compared to students. Youth with high education were three times more likely than students to report mood disorders.

**Table 3:** Socio-demographic predictors of lifetime risk of DSM-IV/CIDI disorders in the SNMHS youth.

	Any lifetime disorder		Anxiety		Mood		Disruptive behavior		Substance use		Eating	
	OR	95% CI	OR	95% CI	OR	95% CI	OR	95% CI	OR	95% CI	OR	95% CI
Sex												
Female	0.8	(0.57–1.12)	1.13	(0.8–1.6)	1.4	(0.87–2.26)	0.47	(0.31–0.71)	0.3	(0.13–0.66)	0.77	(0.44–1.34)
Male	1.00	–	1.00	–	1.00	–	1.00	–	1.00	–	1.00	–
χ^2^_1_	1.71		0.50		1.94		12.88**		8.84*		0.85	
Age												
15–22	1.00	–	1.00	–	1.00	–	1.00	–	1.00	–	1.00	–
23–30	0.79	(0.48–1.3)	1.19	(0.73–1.94)	0.60	(0.29–1.21)	0.85	(0.47–1.53)	0.57	(0.19–1.77)	1.14	(0.48–2.72)
χ^2^_1_	0.84		0.46		2.08		0.30		0.94		0.09	
Education												
Student	1.00	–	1.00	–	1.00	–	1.00	–	1.00	–	1.00	–
Low	2.74	(1.06–7.06)	0.84	(0.33–2.14)	0.43	(0.07–2.78)	6.27	(2.36–16.66)	13.13	(2.54–67.94)	1.98	(0.54–7.25)
Low average	2.21	(0.83–5.89)	2.06	(0.8–5.29)	4.3	(1.3–14.24)	0.4	(0.09–1.85)	36.45	(7.39–179.85)	0.07	(< 0.001–6.29)
High average	1.19	(0.72–1.96)	1.24	(0.76–2.04)	1.41	(0.71–2.82)	1.18	(0.66–2.12)	2.71	(0.97–7.58)	0.54	(0.22–1.32)
High	0.81	(0.43–1.52)	0.78	(0.41–1.48)	3.08	(1.33–7.14)	0.63	(0.28–1.42)	1.44	(0.28–7.46)	1.13	(0.42–3.03)
χ^2^_4_	8.84		5.38		12.36*		21.67**		24.01**		6.63	
Parents' education												
Low	1.00	–	1.00	–	1.00	–	1.00	–	1.00	–	1.00	–
Low average	1.52	(0.93–2.48)	1.85	(1.14–3)	0.53	(0.26–1.1)	1.06	(0.59–1.89)	6.22	(2.16–17.91)	1.25	(0.52–3.04)
High average	1.39	(0.91–2.12)	1.23	(0.79–1.91)	0.82	(0.45–1.49)	1.18	(0.7–1.99)	1.07	(0.31–3.65)	1.81	(0.84–3.91)
High	1.31	(0.77–2.23)	1.31	(0.77–2.25)	0.61	(0.29–1.28)	0.86	(0.44–1.67)	2.21	(0.63–7.7)	1.59	(0.64–3.92)
χ^2^_3_	3.49		6.35		3.65		1.09		16.14*		2.49	
Household income												
Low	1.00	–	1.00	–	1.00	–	1.00	–	1.00	–	1.00	–
Low average	0.55	(0.33–0.93)	0.47	(0.26–0.85)	1.04	(0.49–2.18)	0.33	(0.15–0.72)	0.46	(0.12–1.77)	2.67	(1.29–5.53)
High average	0.87	(0.54–1.41)	1.13	(0.7–1.83)	0.82	(0.39–1.72)	0.54	(0.3–0.98)	0.29	(0.07–1.14)	1.23	(0.53–2.9)
High	0.87	(0.56–1.33)	1.03	(0.67–1.59)	1.22	(0.67–2.21)	0.70	(0.41–1.19)	0.67	(0.25–1.82)	0.93	(0.42–2.05)
χ^2^_3_	4.96		7.72		1.02		10.07*		3.90		8.72*	
Marital status												
Married	1.20	(0.74–1.95)	1.05	(0.64–1.7)	0.73	(0.36–1.47)	1.20	(0.66–2.2)	0.85	(0.25–2.87)	1.25	(0.56–2.8)
Separated/divorced/widowed	3.43	(1.27–9.26)	3.26	(1.29–8.23)	2.23	(0.78–6.34)	4.70	(1.7–12.98)	3.49	(0.63–19.24)	1.63	(0.43–6.2)
Never married	1.00	–	1.00	–	1.00	–	1.00	–	1.00	–	1.00	–
χ^2^_2_	5.93		6.43*		4.11		8.93*		2.47		0.63	
Urbanicity												
Rural	0.64	(0.39–1.05)	1.05	(0.63–1.74)	0.98	(0.47–2.04)	0.92	(0.48–1.76)	0.53	(0.12–2.37)	1.47	(0.63–3.46)
Urban	1.00	–	1.00	–	1.00	–	1.00	–	1.00	–	1.00	–
χ^2^_1_	3.12		0.03		0.00		0.07		0.68		0.79	
Region												
Central	1.00	–	1.00	–	1.00	–	1.00	–	1.00	–	1.00	–
Eastern	1.83	(1.14–2.94)	1.35	(0.84–2.15)	0.59	(0.29–1.2)	2.65	(1.52–4.62)	0.11	(0.02–0.55)	0.88	(0.37–2.09)
Northern	0.99	(0.49–2.01)	0.69	(0.32–1.51)	0.5	(0.15–1.67)	1.75	(0.74–4.14)	0.43	(0.07–2.91)	1.34	(0.4–4.49)
Southern	1.15	(0.66–2)	1.06	(0.6–1.88)	0.6	(0.26–1.39)	1.22	(0.58–2.54)	2.66	(0.83–8.54)	0.48	(0.14–1.65)
Western	1.25	(0.82–1.9)	0.98	(0.64–1.5)	0.89	(0.51–1.58)	0.89	(0.51–1.53)	0.59	(0.24–1.49)	1.55	(0.81–2.96)
χ^2^_4_	6.83		3.32		3.68		16.68*		13.37*		4.97	
Living with parents												
Lived with parents	1.00	–	1.00	–	1.00	–	–	–	–	–	1.00	–
One or both parent died	2.97	(0.63–13.98)	1.63	(0.51–5.2)	0.55	(0.09–3.62)	–	–	–	–	0.93	(0.12–7.17)
Parents divorced	2.31	(0.84–6.31)	1.51	(0.62–3.67)	1.29	(0.41–4.11)	–	–	–	–	2.37	(0.74–7.62)
Parents never lived together/foster care/others	0.28	(0.02–3.8)	0.54	(0.04–7.53)	1.09	(0.05–23.93)	–	–	–	–	1.57	(0.01–450.93)
χ^2^_3_	5.40		1.68		0.58		–		–		2.13	
Family history of disorders												
Yes	3.96	(2.83–5.56)	2.92	(2.09–4.08)	3.02	(1.88–4.84)	4.10	(2.72–6.2)	7.95	(3.21–19.71)	2.60	(1.49–4.54)
No	1.00	–	1.00	–	1.00	–	1.00	–	1.00	–	1.00	–
χ^2^_1_	63.57**		39.51**		20.92**		45.12**		20.01**		11.22**	

Part II sample was used.

*p < 0.05; **p < 0.001.

Any disorder (n = 489); Anxiety (n = 331); Mood (n = 173); Disruptive behavior (n = 173); Substance use (n = 55); Eating (n = 82).

Living with parents was not included in disruptive behavior and substance use disorders due to low frequencies, i.e., for the sub-categories one or both parent died (n ≤ 5), parents divorced (n ≤ 6), and parents never lived together/foster care/others (n = 0).

Individuals with parents who had low average education were six times more likely to be at risk of a substance use disorder than those with parents who had low education. Youth who belonged to households with low average and high average income were significantly less likely compared to those from low income households to have disruptive behavior disorders. However, belonging to a household with low average income doubled the odds of having an eating disorder.

Compared to youth who were never married, individuals who were separated/divorced/widowed had increased risk of anxiety and disruptive behavior disorders. Those who lived in the Eastern region compared to the Central region of the KSA had twice the odds of a disruptive behavior disorder. However, living in the Eastern region was significantly associated with lower rates of substance use disorders compared to the Central region. Finally, Saudi youth with family history of disorders were significantly associated with high lifetime risk of all classes of DSM-IV disorders.

### Lifetime treatment service use

One in seven (14.47%) of Saudi youth with any DSM-IV mental disorder received treatment for a lifetime disorder (Table 4). Treatment was mainly sought from the healthcare sector (9.8%). The majority of the sample did not seek treatment for a lifetime disorder. Youth in the older age group (23–30) were significantly less likely than younger individuals to have received/sought treatment for any DSM-IV mental disorder (Table 5). Those with parents who had high-average education vs. low/low average education were significantly associated with lower odds of seeking/receiving treatment for a lifetime disorder. Individuals with a family history of disorders were 68 times more likely than those without a family history to have received treatment for any lifetime mental disorder. All other associations between the characteristics and lifetime treatment for any DSM-IV disorder were not significant.

**Table 4:** Proportional treatment among youth who received lifetime treatment for DSM-IV/CIDI disorders.

Lifetime treatment	Any lifetime disorder		
	N	%	SE
Any healthcare	69	9.80	2.07
Any non-healthcare	46	6.52	1.61
Any treatment	101	14.47	2.66
No treatment	400	85.53	2.66

Part II Sample was used (n = 971); any disorder (n = 501).

**Table 5:** Predictors of treatment among Saudi youth with DSM IV/CIDI lifetime mental disorders.

	Any lifetime disorder	
	OR	95% CI
Sex		
Female	1.12	(0.21–5.9)
Male	1.00	–
χ^2^_1_	0.02	
Age		
15–22	1.00	–
23–30	0.06	(0.01–0.73)
χ^2^_1_	4.86*	
Education		
Student	1.00	–
Low/low average	1.29	(0.08–21.94)
High average	2.31	(0.27–20.17)
High	2.41	(0.17–35.24)
χ^2^_3_	0.70	
Parents' education		
Low/low average	1.00	–
High average	0.07	(0.01–0.56)
High	2.52	(0.22–29.25)
χ^2^_2_	8.98*	
Household income		
Low	1.00	–
Low average	0.54	(0.05–6.37)
High average	5.77	(0.53–62.92)
High	2.53	(0.32–20.13)
χ^2^_3_	3.28	
Marital status		
Married/separated/divorced/widowed	6.83	(0.93–50.33)
Never married	1.00	–
χ^2^_1_	3.56	
Urbanicity		
Rural	2.62	(0.21–32.14)
Urban	1.00	–
χ^2^_1_	0.57	
Region		
Central	1.00	–
Eastern	0.22	(0.01–3.36)
Northern	0.43	(0.02–11.73)
Southern	0.07	(0–1.26)
Western	0.22	(0.03–1.87)
χ^2^_4_	4.13	
Family history of disorders		
Yes	68.71	(7.58–622.52)
No	1.00	–
χ^2^_1_	14.15**	

Part II sample was used; *p < 0.05; **p < 0.001.

Any disorder (n = 101), out of the respondents who received any treatment.

Low and low average categories were combined for education and parents' education due to low frequencies (n ≤ 10); married/separated/widowed/divorced were combined for marital status due to low frequencies for the subcategory separated/divorced/widowed (n < 10).

## Discussion

This study presents the lifetime prevalence and treatment estimates on a range of mental disorders in a nationally representative household sample of Saudi youth. Although the prevalence of any lifetime DSM-IV mental disorder in this study is higher than the rate reported in previous global studies^1^, and an Omani study^24^, it is similar to the rates summarized in a systematic review and meta-analysis of studies from the GCC^19^, and the lifetime mental disorder prevalence among youth in the United States (US)^12^, and Finland^67^. Moreover, prior estimates may be underestimated given evidence that mental disorders are increasing^68–71^.

The SNMHS assessed various classes of DSM-IV disorders and their subtypes compared to previous national Saudi literature that focused on specific disorders^46,47^. Using the SNMHS dataset, we found that anxiety disorders were the most prevalent class of DSM-IV disorders among Saudi youth, followed by disruptive behavior disorders, mood disorders and eating disorders. The rates for commonly prevalent specific disorders classified under these classes of DSM-IV disorders—such as generalized anxiety disorders, social phobia, major depressive disorder, and disordered eating behaviors) were consistent with previous Saudi^43–45,47^ and GCC studies^19,21^. However, among the diagnostic surprises were the high rates of separation anxiety disorder and ADHD (14.69% and 11.29%). These rates are higher than in neighboring countries, for example, Oman^24^. Because this is the first such study in KSA, replication of prevalence and data across the full age spectrum is needed. We intend to address these specific diagnoses in further detail in additional reports.

Regarding significant sex differences related to disorder-specific prevalence found in our study, these were in line with previous Western and Arab literature indicating that social phobia^72,73^, obsessive–compulsive disorder^74,75^, and major depressive disorder^24,48,67^ affect females more than males. Previous Western evidence also suggests that young adults aged 18 and older have high prevalence of post-traumatic stress disorder^76,77^. Consistent with this, the older age group (23–30) of Saudi youth had higher rates of posttraumatic stress disorder in comparison to younger individuals. As observed previously^12^, the rates of emotional and behavior disorders are comparable, and for some specific disorders even higher than the major physical conditions prevalent among youth, such as the national rates of asthma^78^ or diabetes^79^. Yet mental disorders are still not regarded as seriously both globally and locally within the public health context.

With respect to sociodemographic correlates of lifetime mental disorder, we found that females were less likely to have disruptive behavior disorders and substance use disorders in line with previous Western^67,80–82^ and Arab studies^21,83^. However, this may be because females present with more subtle expressions of hyperactivity and impulsivity, or altogether different developmental pathways suggestive of disruptive behavior disorder prevalence^84,85^. Indeed, some have raised criticism and made a case for inclusion of more female-sensitive items for ADHD and conduct disorder in the DSM-III and DSM-IV respectively, which have better descriptors of symptoms in boys than in girls^86,87^. Additionally, although drug use may be prevalent among Saudi youth^83^, data are limited on drug use among females^88^ due to the conservative nature of the society and gender segregation, potentially making drugs less accessible to females than males in the KSA.

Compared to those who were students at the time, Saudi youth who reported having lower educational attainment (low and low-average education) were associated with increased risk of lifetime disruptive behavior disorders, mood disorders and substance use disorders. These findings were consistent with previous work on youth from the US, Finland and New Zealand concerning educational attainment and risk of these lifetime mental disorders^67,89–91^. However, our study also found that youth with high educational attainment had three times the odds of mood disorders compared to those enrolled as students at the time. These mixed findings are supported by previous work^92^ indicating that the increased risk of mood disorders—such as major depressive disorder—must be considered within the context of a young person’s life history, their social, familial, personal circumstances, as such varied and individual factors may confound the linkage between adverse outcomes—such as educational underachievement—and increased risk of depression.

Parental education (low-average vs low) was also significantly associated with increased risk of lifetime substance use disorders among Saudi youth. This finding conflicted with US studies^93,94^ but aligned with a Lebanese study^28^ that suggested parental education was a significant correlate of having a psychiatric disorder (30 day-prevalence). Additionally, household income (low average and high average vs low) was significantly associated with lower rates of a disruptive behavior disorder, consistent with Arab studies on ADHD prevalence^21^. However, the association between household income and the risk of disruptive behavior disorder such as conduct disorder is not straightforward as trends in income inequalities only account for a small fraction of rise in conduct problems among youth^95,96^.

Although a previous study reported no association between household income and lifetime eating disorder among American adolescents^97^, we found that Saudi youth from households with low average income had increased risk of an eating disorder compared to those from low income households. This may be due to the growing industrialization in the KSA and consequent Western influence on the society, which has been reported to increase the risk of eating disorders in Arabs^98^.

In contrast to a Canadian study^99^, Saudi youth who were separated/divorced/widowed had increased rates of lifetime anxiety disorders compared to those who were never married. Additionally, some evidence from the US^100^ supported the significant association between being separated, divorced, or widowed and the increased risk of a disruptive behavior disorder among Saudi youth.

The significant association between region (Eastern vs Central) and increased risk of a disruptive behavior disorder among Saudi youth was supported by earlier studies on ADHD prevalence but with the limitation that previous estimates from these regions were almost comparable (3.5% vs 3.4%), potentially due to differing diagnostic methods and corresponded only to primary school children^101,102^. Consistent with adolescent studies from the US^103^, Austria^104^, and Lebanon^28^, family history of psychiatric disorders significantly increased the risk of all classes of mental disorders among Saudi youth.

Lifetime service use rates (14.47%) among Saudi youth were similar to those from Oman (5.2–13.2%)^23^. Given that mental health care services in the KSA are largely based within the traditional health sector^105^, most youth received or sought treatment through the health sector in our study. However, the majority (85.53%) did not receive/seek lifetime treatment for any mental disorder, consistent with the US adolescent study^106^. Arab adolescents tend to seek help through their social networks or traditional healers^20^. Additionally, web-based mental health resources are perceived as facilitators of help-seeking among young adults in the KSA, as these allow anonymity and stigma avoidance^107^.

Significant findings on age (aged 23–30 vs. aged 15–22) and parental education levels (high average vs. low) emerging as possible barriers to seeking/receiving lifetime treatment among Saudi youth contrasted with previous American and Mexican studies on age and lifetime treatment^106^, and parental education and 12-month treatment^108,109^. There was, however, some evidence from North America^110,111^ to support family history of disorders as a significant predictor of seeking lifetime treatment among Saudi youth.

Results of this study must be interpreted in light of its limitations. First, causality cannot be implied by any of the significant associations found in our cross-sectional study. Second, respondents’ retrospective recall—subject to error and bias—in addition to stigma attached to reporting mental health concerns^107,112^, may have led to underestimates of lifetime prevalence^113^. Third, some segments of the population such as those who are institutionalized or do not speak Arabic were excluded from the SNMHS sample^59^; the under-representation of these groups may also have led to lower prevalence and treatment estimates. Fourth, the diagnostic nomenclature for youth (as used in this study) can be problematic as young people undergo a natural period of emotional, behavioral, and physical change^12^. The current system does not consider the plasticity of adolescent development. However, precluding the debate surrounding diagnostic criteria, our findings offer a glimpse for the first time into a wide range of mental health conditions prevalent among Saudi youth that need immediate public health attention. Effective and culturally appropriate screening, prevention, and intervention approaches using evidence-based guidelines for youth are required^25^. Our findings related to prevalence, treatment, and their significant correlates can inform future policies, public health planning and research in the KSA and the GCC. There is now a need for a prospective study that can examine in-depth mental disorders’ onset during adolescence, and the disorders’ trajectory into adulthood.

## Data Availability

Please contact the corresponding author for data requests.

## References

[1] Polanczyk GV, Salum GA, Sugaya LS, Caye A, Rohde LA (2015). Annual research review: A meta-analysis of the worldwide prevalence of mental disorders in children and adolescents. J. Child Psychol. Psychiatry.

[2] Racine N (2021). Global prevalence of depressive and anxiety symptoms in children and adolescents during COVID-19: A meta-analysis. JAMA Pediatr..

[3] Patton GC (2016). Our future: A lancet commission on adolescent health and wellbeing. Lancet.

[4] General Authority for Statistics. *Saudi Youth in Numbers: A Report for International Youth Day 2020*. (2020).

[5] Programme, U. N. D. (United Nations Development Programme, Regional Bureau for Arab States New York, 2016).

[6] Liang L (2020). The effect of COVID-19 on youth mental health. Psychiatr. Q..

[7] Blakemore S-J, Mills KL (2014). Is adolescence a sensitive period for sociocultural processing?. Annu. Rev. Psychol..

[8] Carey CE (2016). Associations between polygenic risk for psychiatric disorders and substance involvement. Front. Genet..

[9] Kessler RC (2007). Lifetime prevalence and age-of-onset distributions of mental disorders in the World Health Organization's world mental health survey initiative. World Psychiatry.

[10] Raballo A, Poletti M, McGorry P (2017). Architecture of change: Rethinking child and adolescent mental health. Lancet Psychiatry.

[11] Vohs KD, Piquero AR (2021). Self-control at 220 miles per hour: Steering and braking to achieve optimal outcomes during adolescence. Curr. Dir. Psychol. Sci..

[12] Merikangas KR (2010). Lifetime prevalence of mental disorders in US adolescents: Results from the national comorbidity survey replication-adolescent supplement (NCS-A). J. Am. Acad. Child Adolesc. Psychiatry.

[13] Vaillant GE (2003). Mental health. Am. J. Psychiatry.

[14] Park MJ, PaulMulye T, Adams SH, Brindis CD, Irwin CE (2006). The health status of young adults in the United States. J. Adolescent Health.

[15] Slater CL (2003). Generativity versus stagnation: An elaboration of Erikson's adult stage of human development. J. Adult Dev..

[16] Barker DJ (2003). The developmental origins of adult disease. Eur. J. Epidemiol..

[17] Leckman JF, Leventhal BL (2008). Editorial: A global perspective on child and adolescent mental health. J. Child Psychol. Psychiatry.

[18] Costello EJ, Mustillo S, Erkanli A, Keeler G, Angold A (2003). Prevalence and development of psychiatric disorders in childhood and adolescence. Arch. Gen. Psychiatry.

[19] Chan MF (2021). Child and adolescent mental health disorders in the GCC: A systematic review and meta-analysis. Int. J. Pediatr. Adolesc. Med..

[20] Dardas LA, Bailey DE, Simmons LA (2016). Adolescent depression in the Arab region: A systematic literature review. Issues Ment. Health Nurs..

[21] Farah LG (2009). ADHD in the arab world: A review of epidemiologic studies. J. Atten. Disord..

[22] Musaiger AO (2013). Risk of disordered eating attitudes among adolescents in seven Arab countries by gender and obesity: A cross-cultural study. Appetite.

[23] Al Riyami AA, Al Adawi SH, Al Kharusi HA, Morsi MM, Jaju SS (2009). Health services utilization by school going Omani adolescents and youths with DSM IV mental disorders and barriers to service use. Int. J. Ment. Health Syst..

[24] Jaju S, Al-Adawi S, Al-Kharusi H, Morsi M, Al-Riyami A (2009). Prevalence and age-of-onset distributions of DSM IV mental disorders and their severity among school going Omani adolescents and youths: WMH-CIDI findings. Child Adolesc. Psychiatry Ment. Health.

[25] Dardas LA, Silva SG, Smoski MJ, Noonan D, Simmons LA (2018). The prevalence of depressive symptoms among Arab adolescents: Findings from Jordan. Public Health Nurs..

[26] Ismayilova L, Hmoud O, Alkhasawneh E, Shaw S, El-Bassel N (2013). Depressive symptoms among Jordanian youth: Results of a national survey. Community Ment. Health J..

[27] Schulte SJ (2016). Predictors of binge eating in male and female youths in the United Arab Emirates. Appetite.

[28] Maalouf FT (2016). Psychiatric disorders among adolescents from Lebanon: Prevalence, correlates, and treatment gap. Soc. Psychiatry Psychiatr. Epidemiol..

[29] Al Makadma AS (2017). Adolescent health and health care in the Arab Gulf countries: Today's needs and tomorrow's challenges. Int. J. Pediatr. Adolesc. Med..

[30] Dick B, Ferguson BJ (2015). Health for the world's adolescents: A second chance in the second decade. J. Adolesc. Health.

[31] Fakhr El-Islam M (2008). Arab culture and mental health care. Transcult. Psychiatry.

[32] Obermeyer CM, Bott S, Sassine AJ (2015). Arab adolescents: Health, gender, and social context. J. Adolesc. Health.

[33] UNICEF. *The Situation of Adolescents and Youth in the Middle East & North Africa Region: A Desk Review of Data on Current Trends and Emerging Issues*. (UNICEF MENA Regional Office, 2013).

[34] General Authority for Statistics. Population mid-year 2021. (2021).

[35] General Authority for Statistics. *Population Estimates by Gender and Age GROUPs*. (2021).

[36] General Authority for Statistics. *Saudi Population Estimated Median Age*. (2021).

[37] Al-Sughayr AM, Ferwana MS (2012). Prevalence of mental disorders among high school students in National Guard Housing, Riyadh, Saudi Arabia. J. Fam. Community Med..

[38] Asal A-RA, Abdel-Fattah MM (2007). Prevalence, symptomatology, and risk factors for depression among high school students in Saudi Arabia. Neurosci. J..

[39] Mahfouz AA (2009). Adolescents' mental health in Abha City, Southwestern Saudi Arabia. Int. J. Psychiatry Med..

[40] Al Gelban KS (2009). Prevalence of psychological symptoms in Saudi secondary school girls in Abha, Saudi Arabia. Ann. Saudi Med..

[41] Al-Gelban KS, Al-Amri HS, Mostafa OA (2009). Prevalence of depression, anxiety and stress as measured by the depression, anxiety, and stress scale (DASS-42) among secondary school girls in Abha, Saudi Arabia. Sultan Qaboos Univ. Med. J..

[42] Al-Qahtani, A. M. & Al-Harbi, M. B. *Prevalence and Risk Factors of Anxiety Among Female Governmental Secondary Schools Students in Al-madinah, Saudi Arabia*. (2017).

[43] Fatima W, Ahmad LM (2018). Prevalence of disordered eating attitudes among adolescent girls in Arar City, Kingdom of Saudi Arabia. Health Psychol. Res..

[44] Al-Gelban KS (2007). Depression, anxiety and stress among Saudi adolescent school boys. J. R. Soc. Promot. Health.

[45] Ghazwani JY, Khalil SN, Ahmed RA (2016). Social anxiety disorder in Saudi adolescent boys: Prevalence, subtypes, and parenting style as a risk factor. J. Fam. Community Med..

[46] Al Buhairan F (2017). The relationship of bullying and physical violence to mental health and academic performance: A cross-sectional study among adolescents in Kingdom of Saudi Arabia. Int. J. Pediatr. Adolesc. Med..

[47] AlBuhairan FS (2015). Time for an adolescent health surveillance system in Saudi Arabia: Findings From “Jeeluna”. J. Adolesc. Health.

[48] Abou Abbas O, AlBuhairan F (2017). Predictors of adolescents’ mental health problems in Saudi Arabia: Findings from the Jeeluna® national study. Child. Adolesc. Psychiatry Ment. Health.

[49] Altwaijri YA (2020). Lifetime prevalence and age-of-onset distributions of mental disorders in the Saudi National mental health survey. Int. J. Methods Psychiatr. Res..

[50] Mahsoon A (2020). Parental support, beliefs about mental illness, and mental help-seeking among young adults in Saudi Arabia. Int. J. Environ. Res. Public Health.

[51] Patel V, Flisher AJ, Hetrick S, McGorry P (2007). Mental health of young people: A global public-health challenge. Lancet.

[52] Gearing RE (2015). Stigma and mental health treatment of adolescents with depression in Jordan. Community Ment. Health J..

[53] Vicente B (2012). Prevalence of child and adolescent psychiatric disorders in Santiago, Chile: A community epidemiological study. Soc. Psychiatry Psychiatr. Epidemiol..

[54] World Health Organization (2011). Atlas: Child, Adolescent and Maternal Mental Health Resources in the Eastern Mediterranean Region.

[55] Heeringa, S. *et al.* Sample designs and sampling procedures. In *The WHO World Mental Health Surveys: Global Perspectives on the Epidemiology of Mental Disorders*, 14–32 (2008).

[56] Mneimneh ZN, Heeringa SG, Lin Y-C, Altwaijri YA, Nishimura R (2020). The Saudi national mental health survey: Sample design and weight development. Int. J. Methods Psychiatr. Res..

[57] Harkness, J. *et al.* Translation procedures and translation assessment in the World Mental Health Survey Initiative. In *The WHO World Mental Health Surveys: Global Perspectives on the Epidemiology of Mental Disorders*, 91–113 (2008).

[58] Shahab M (2019). Implementing the TRAPD model for the Saudi adaptation of the World Mental Health composite international diagnostic interview 3.0. Int. J. Ment. Health Syst..

[59] Altwaijri YA (2020). The Saudi national mental health survey: Survey instrument and field procedures. Int. J. Methods Psychiatr. Res..

[60] Pennell, B. *et al.* Implementation of the world mental health surveys. In *The WHO World Mental Health Surveys: Global Perspectives on the Epidemiology of Mental Disorders*, 33–57 (2008).

[61] Hyder S (2017). Evidence-based guideline implementation of quality assurance and quality control procedures in the Saudi National Mental Health Survey. Int. J. Ment. Health Syst..

[62] Kessler RC, Üstün TB (2004). The world mental health (WMH) survey initiative version of the World Health Organization (WHO) composite international diagnostic interview (CIDI). Int. J. Methods Psychiatr. Res..

[63] Association, A. P. *Diagnostic and Statistical Manual of Mental Disorders, Text Revision (DSM-IV-TR®)*. (2010).

[64] Haro JM (2006). Concordance of the composite international diagnostic interview version 3.0 (CIDI 3.0) with standardized clinical assessments in the WHO world mental health surveys. Int. J. Methods Psychiatr. Res..

[65] Kessler RC (2020). Clinical reappraisal of the composite international diagnostic interview version 3.0 in the Saudi national mental health survey. Int. J. Methods Psychiatr. Res..

[66] Al-Subaie AS (2020). Lifetime treatment of DSM-IV mental disorders in the Saudi national mental health survey. Int. J. Methods Psychiatr. Res..

[67] Suvisaari J (2009). Mental disorders in young adulthood. Psychol. Med..

[68] Cybulski L (2021). Temporal trends in annual incidence rates for psychiatric disorders and self-harm among children and adolescents in the UK, 2003–2018. BMC Psychiatry.

[69] Deighton J (2019). Prevalence of mental health problems in schools: Poverty and other risk factors among 28 000 adolescents in England. Br. J. Psychiatry.

[70] Keyes KM, Gary D, O’Malley PM, Hamilton A, Schulenberg J (2019). Recent increases in depressive symptoms among US adolescents: Trends from 1991 to 2018. Soc. Psychiatry Psychiatr. Epidemiol..

[71] Patalay P, Gage SH (2019). Changes in millennial adolescent mental health and health-related behaviours over 10 years: A population cohort comparison study. Int. J. Epidemiol..

[72] Furmark T (1999). Social phobia in the general population: Prevalence and sociodemographic profile. Soc. Psychiatry Psychiatr. Epidemiol..

[73] Wittchen HU, Stein MB, Kessler RC (1999). Social fears and social phobia in a community sample of adolescents and young adults: Prevalence, risk factors and co-morbidity. Psychol. Med..

[74] Vivan ADS (2013). Obsessive-compulsive symptoms and obsessive-compulsive disorder in adolescents: a population-based study. Braz. J. Psychiatry.

[75] Fawcett EJ, Power H, Fawcett JM (2020). Women are at greater risk of OCD than men: A meta-analytic review of OCD prevalence worldwide. J. Clin. Psychiatry.

[76] de Vries G-J, Olff M (2009). The lifetime prevalence of traumatic events and posttraumatic stress disorder in the Netherlands. J. Trauma. Stress.

[77] Lewis SJ (2019). The epidemiology of trauma and post-traumatic stress disorder in a representative cohort of young people in England and Wales. Lancet Psychiatry.

[78] Musharrafieh U, Tamim H, Houry R, AlBuhairan F (2020). A nationwide study of asthma correlates among adolescents in Saudi Arabia. Asthma Res. Pract..

[79] Al-Rubeaan K (2015). National surveillance for type 1, type 2 diabetes and prediabetes among children and adolescents: A population-based study (SAUDI-DM). J. Epidemiol. Community Health.

[80] Knopf D, Park MJ, Mulye TP (2008). The Mental Health of Adolescents: A National Profile.

[81] Moffitt TE, Caspi A, Rutter M, Silva PA (2001). Sex Differences in Antisocial Behaviour: Conduct Disorder, Delinquency, and Violence in the Dunedin Longitudinal Study.

[82] Offord DR, Boyle MH, Racine YA (1991). The epidemiology of antisocial behavior in childhood and adolescence. Dev. Treatm. Child. Aggress..

[83] Saquib N, Rajab AM, Saquib J, AlMazrou A (2020). Substance use disorders in Saudi Arabia: A scoping review. Subst. Abuse Treatm. Prev. Policy.

[84] Loeber R, Capaldi DM, Costello E (2013). Disruptive Behavior Disorders.

[85] Rucklidge JJ (2010). Gender differences in attention-deficit/hyperactivity disorder. Psychiatr. Clin. N. Am..

[86] Ohan JL, Johnston C (2005). Gender appropriateness of symptom criteria for attention-deficit/hyperactivity disorder, oppositional-defiant disorder, and conduct disorder. Child Psychiatry Hum. Dev..

[87] Zoccolillo M (1993). Gender and the development of conduct disorder. Dev. Psychopathol..

[88] Khalawi A, Ibrahim A, Alghamdi A (2017). Risk factors potentiating substance abuse among Saudi females: A case-control study. Int. J. Med. Res. Prof..

[89] Mojtabai R (2015). Long-term effects of mental disorders on educational attainment in the National Comorbidity Survey ten-year follow-up. Soc. Psychiatry Psychiatr. Epidemiol..

[90] Schepis TS, Teter CJ, McCabe SE (2018). Prescription drug use, misuse and related substance use disorder symptoms vary by educational status and attainment in US adolescents and young adults. Drug Alcohol Depend..

[91] Fergusson DM, Boden JM (2008). Cannabis use and later life outcomes. Addiction.

[92] Fergusson DM, Woodward LJ (2002). Mental health, educational, and social role outcomes of adolescents with depression. Arch. Gen. Psychiatry.

[93] Haller M, Handley E, Chassin L, Bountress K (2010). Developmental cascades: Linking adolescent substance use, affiliation with substance use promoting peers, and academic achievement to adult substance use disorders. Dev. Psychopathol..

[94] Patrick ME, Schulenberg JE, Omalley PM, Johnston LD, Bachman JG (2011). Adolescents' reported reasons for alcohol and marijuana use as predictors of substance use and problems in adulthood. J. Stud. Alcohol Drugs.

[95] Collishaw S, Goodman R, Pickles A, Maughan B (2007). Modelling the contribution of changes in family life to time trends in adolescent conduct problems. Soc. Sci. Med..

[96] Marmot M, Wilkinson RG (2001). Psychosocial and material pathways in the relation between income and health: A response to Lynch et al. BMJ.

[97] Swanson SA, Crow SJ, Le Grange D, Swendsen J, Merikangas KR (2011). Prevalence and correlates of eating disorders in adolescents: Results from the national comorbidity survey replication adolescent supplement. Arch. Gen. Psychiatry.

[98] Melisse B, de Beurs E, van Furth EF (2020). Eating disorders in the Arab world: A literature review. J. Eat. Disord..

[99] Nguyen CT, Fournier L, Bergeron L, Roberge P, Barrette G (2005). Correlates of depressive and anxiety disorders among young Canadians. Can. J. Psychiatry.

[100] Olino TM, Seeley JR, Lewinsohn PM (2010). Conduct disorder and psychosocial outcomes at age 30: Early adult psychopathology as a potential mediator. J. Abnorm. Child Psychol..

[101] Albatti TH (2017). Prevalence of attention deficit hyperactivity disorder among primary school-children in Riyadh, Saudi Arabia; 2015–2016. Int. J. Pediatr. Adolesc. Med..

[102] Jenahi E, Khalil MS, Bella H (2012). Prevalence of attention deficit hyperactivity symptoms in female schoolchildren in Saudi Arabia. Ann. Saudi Med..

[103] McLaughlin KA (2012). Childhood adversities and first onset of psychiatric disorders in a national sample of US adolescents. Arch. Gen. Psychiatry.

[104] Wagner G (2017). Mental health problems in Austrian adolescents: A nationwide, two-stage epidemiological study applying DSM-5 criteria. Eur. Child Adolesc. Psychiatry.

[105] Al-Subaie AS, Al-Habeeb A, Altwaijri YA (2020). Overview of the Saudi national mental health survey. Int. J. Methods Psychiatric Res..

[106] Merikangas KR (2011). Service utilization for lifetime mental disorders in US adolescents: Results of the national comorbidity survey-adolescent supplement (NCS-A). J. Am. Acad. Child Adolesc. Psychiatry.

[107] Noorwali R (2022). Barriers and facilitators to mental health help-seeking among young adults in Saudi Arabia: A qualitative study. Int. J. Environ. Res. Public Health.

[108] Benjet C, Borges G, Medina-Mora ME, Zambrano J, Aguilar-Gaxiola S (2009). Youth mental health in a populous city of the developing world: Results from the Mexican adolescent mental health survey. J. Child Psychol. Psychiatry.

[109] Olfson M, He J-P, Merikangas KR (2013). Psychotropic medication treatment of adolescents: Results From the National comorbidity survey-adolescent supplement. J. Am. Acad. Child Adolesc. Psychiatry.

[110] Cornelius JR, Pringle J, Jernigan J, Kirisci L, Clark DB (2001). Correlates of mental health service utilization and unmet need among a sample of male adolescents. Addict. Behav..

[111] Shapiro J (2014). Correlates of psychiatric hospitalization in a clinical sample of Canadian adolescents with bipolar disorder. Compr. Psychiatry.

[112] Alattar N, Felton A, Stickley T (2021). Mental health and stigma in Saudi Arabia: a scoping review. Ment. Health Rev. J..

[113] Kruijshaar ME (2005). Lifetime prevalence estimates of major depression: An indirect estimation method and a quantification of recall bias. Eur. J. Epidemiol..

